# LSTrAP-Crowd: prediction of novel components of bacterial ribosomes with crowd-sourced analysis of RNA sequencing data

**DOI:** 10.1186/s12915-020-00846-9

**Published:** 2020-09-03

**Authors:** Benedict Hew, Qiao Wen Tan, William Goh, Jonathan Wei Xiong Ng, Marek Mutwil

**Affiliations:** School of Biological Sciences, Nanyang Technological University, 60 Nanyang Drive, Singapore, 637551 Singapore

**Keywords:** Co-expression, Bacteria, Ribosome, Protein synthesis, RNA-seq, Crowdsourcing

## Abstract

**Background:**

Bacterial resistance to antibiotics is a growing health problem that is projected to cause more deaths than cancer by 2050. Consequently, novel antibiotics are urgently needed. Since more than half of the available antibiotics target the structurally conserved bacterial ribosomes, factors involved in protein synthesis are thus prime targets for the development of novel antibiotics. However, experimental identification of these potential antibiotic target proteins can be labor-intensive and challenging, as these proteins are likely to be poorly characterized and specific to few bacteria. Here, we use a bioinformatics approach to identify novel components of protein synthesis.

**Results:**

In order to identify these novel proteins, we established a Large-Scale Transcriptomic Analysis Pipeline in Crowd (LSTrAP-Crowd), where 285 individuals processed 26 terabytes of RNA-sequencing data of the 17 most notorious bacterial pathogens. In total, the crowd processed 26,269 RNA-seq experiments and used the data to construct gene co-expression networks, which were used to identify more than a hundred uncharacterized genes that were transcriptionally associated with protein synthesis. We provide the identity of these genes together with the processed gene expression data.

**Conclusions:**

We identified genes related to protein synthesis in common bacterial pathogens and thus provide a resource of potential antibiotic development targets for experimental validation. The data can be used to explore additional vulnerabilities of bacteria, while our approach demonstrates how the processing of gene expression data can be easily crowd-sourced.

## Background

Bacterial resistance to antibiotics is a serious and growing concern in public health, taking ca. 99,000 lives and costing 21–34 billion USD per year in the USA [1]. Methicillin-resistant Gram-positive *Staphylococcus aureus* (MRSA) and Gram-negative *Pseudomonas aeruginosa* are the leading causes of serious infections as they form biofilms. Biofilms are complex bacterial communities embedded in an extracellular matrix, and these communities are able to resist antimicrobial agents [2]. For instance, bacteria can be up to 1000× more tolerant to antibiotics when they grow as a biofilm, compared to single-cell suspension (planktonic cells). Consequently, new antibiotics are urgently needed to combat these resistance mechanisms, either alone or in combination with existing drugs.

More than half of the antibiotics currently in use target the bacterial ribosome, typically at the elongation step of protein synthesis [3], through direct or proximal binding of the peptidyl transferase center (PTC) which catalyzes peptide bond formation [4]. PTC-targeting antibiotics (e.g., lincosamides, pleuromutilins, chloramphenicol, and group A streptogramins), inhibit protein synthesis by obstructing the proper positioning of the tRNA substrates [5].

Bacteria can be intrinsically less sensitive to antibiotics due to less efficient uptake of antibiotics or mutations in ribosomal proteins that result in decreased drug-binding efficiency [3, 6]. The most frequently encountered acquired resistance mechanism involves the methylation of the ribosomal RNA (e.g., by Erm family methyltransferases), which results in decreased drug-binding efficiency and increased viability in the presence of antibiotics [7, 8]. As modification of the ribosomes can result in a decrease in fitness, these methyltransferases genes tend to be induced by the relevant antibiotics through translation attenuation [9, 10]. Alternatively, the antibiotics can also be modified, pumped out, or degraded, thus lowering the intracellular concentration to non-toxic levels [3, 11]. Another mechanism is ribosome protection, where the antibiotic is actively dislodged from the ribosome by ATP-binding cassette F (ABC-F) protein, as observed in many clinical isolates (e.g., *Pseudomonas aeruginosa, Escherichia coli, Staphylococcus aureus*, *Enterococcus faecalis* and *Listeria monocytogenes*) [12–14].

While the structure of the ribosomes is well conserved, structural features of ribosomes may vary significantly between different species, suggesting species-specific adaptations of protein synthesis [15–23]. For example, structural analysis of mycobacterial ribosome revealed that the 30S ribosomal subunit lacks the protein bS21 that is found in *Escherichia coli*. Instead, the mycobacteria employ a unique protein bS22 near the decoding center (DC), thereby keeping the overall number of ribosomal proteins in 30S subunit the same as in *E. coli* [19]. Thus, the identification of novel bacteria ribosomal components has great potential for the development of species-specific antibiotics. However, the identification of these novel components using traditional molecular or structural biology approaches is time-consuming.

Bioinformatic approaches are used to predict gene function and can be used to identify novel components of protein synthesis. Newly sequenced genomes of all organisms are typically first annotated using sequence similarity analysis, where the genes are annotated based on the DNA/protein sequence similarity to characterized genes/proteins [24]. While sequence similarity analysis is well established and gives a quick overview of gene functions in a new genome, it has its caveats as genes can (i) have multiple functions, (ii) sub- or neo-functionalize, and/or (iii) have no sequence similarity to characterized genes. Thus, while sequence similarity analysis is a powerful method, it requires other methods to complement it [24, 25].

The wide availability of RNA sequencing (RNA-seq) data makes it possible to study gene function from the perspective of gene expression [24, 26–28]. Co-expression analysis is based on the observation that genes that have similar expression profiles across experiments tend to be functionally related [24, 25, 29]. These co-expressed genes can be identified by analyzing publicly available microarrays or RNA-seq data, and the co-expression relationships can be represented as networks. In a co-expression network, genes are represented as nodes, where edges connect co-expressed nodes (links) [30–40]. The networks can be mined for groups of highly connected genes (called clusters or modules) that likely represent genes that are involved in the same biological process. Due to the ubiquity of expression data, and the ability to complement DNA/protein sequence-based gene function prediction approaches, coexpression networks have become a popular tool to elucidate the function of genes. The networks have predicted the function of genes involved in a wide range of processes, such as various cellular processes [38, 41–43], transcriptional regulation [44], physiological responses to the environment and stress [45, 46], and the biosynthesis of metabolites [34, 47–49].

The amount of gene expression data has expanded vastly over the last decade, resulting in > 1000-fold increase in nucleotide bases on NCBI Sequence Read Archive (SRA), from 11 TB (2010) to 12 PB (2020). Due to limitations in software used to estimate gene expression from RNA-seq data, analyzing all this data would have been unthinkable a decade ago. However, drastic improvements to the speed and efficiency of software, such as Kallisto [50] and salmon [51], allow the analysis of gigabytes of data on even a Raspberry Pi-like miniature computer [48]. Recently, combined the availability of cloud computing and the user-friendliness of the Jupyter notebooks to implement a large-scale transcriptomic analysis pipeline, LSTrAP-Cloud [47]. Importantly, though Google Colab, the pipeline gives access to a free cloud computer with 2 Xeon cores, with at least 15 GB of permanent storage (as provided by users Google drive account) and 12 GB of RAM, giving biologists both the software and hardware to perform large-scale co-expression analysis.

In this study, we introduce Large-Scale Transcriptomic Analysis Pipeline in Crowd (LSTrAP-Crowd). This simple pipeline was used by 285 undergraduate students to process RNA-seq data of some of the 17 most notorious bacterial pathogens. Within a week, the students processed 26,269 RNA-seq samples, comprising 263,757,103,900 (~ 263 billion) reads and 26.38 terabytes of data. The gene expression data was used to construct co-expression networks, which were mined for the presence of uncharacterized genes that were co-expressed with the bacterial ribosomes. In total, we have predicted more than 100 putative proteins to be involved in protein synthesis in the 17 bacterial pathogens.

## Results

### Obtaining and quality-controlling gene expression data for 17 bacterial pathogens

In this study, we analyzed the gene expression data of 17 notorious bacterial pathogens that cause numerous diseases, such as pharyngitis, tonsillitis, scarlet fever, cellulitis, erysipelas, rheumatic fever, post-streptococcal glomerulonephritis, necrotizing fasciitis, and many others (Table 1). While more bacterial pathogens were considered, we only analyzed bacteria that had at least 100 RNA-seq samples based on Illumina technology found in the Sequence Read Archive [52]. In total, 26,269 RNA-seq samples were analyzed.

**Table 1:** Genomic properties of the 17 bacteria and the RNA-seq sample statistics

Bacteria	Number of genes	Genome size (Mb)	Student groups analyzing the data	RNA-seq samples: passed QC/total
*Campylobacter jejuni*	1635	1.65	1	219/320
*Clostridioides difficile*	3769	4.27	1	182/381
*Enterococcus faecalis*	2579	3.11	1	81/195
*Escherichia coli*	4141	5.44	13	2494/7154
*Haemophilus influenzae*	2098	1.79	2	189/448
*Helicobacter pylori*	1720	1.62	1	84/167
*Klebsiella pneumoniae*	5141	5.78	2	536/639
*Listeria monocytogenes*	2817	2.78	2	370/572
*Mycobacterium tuberculosis*	4023	4.33	11	2414/6495
*Mycoplasma pneumoniae*	629	0.82	3	365/956
*Neisseria gonorrhoeae*	2159	2.15	1	102/371
*Pseudomonas aeruginosa*	6512	6.09	7	1372/2662
*Salmonella enterica*	4554	4.79	3	611/1284
*Staphylococcus aureus*	2638	2.90	5	1112/1963
*Streptococcus pneumoniae*	2043	2.13	2	422/647
*Streptococcus pyogenes*	1660	1.85	4	1340/1705
*Vibrio cholerae*	3648	4.00	1	167/311

**Fig. 1:**
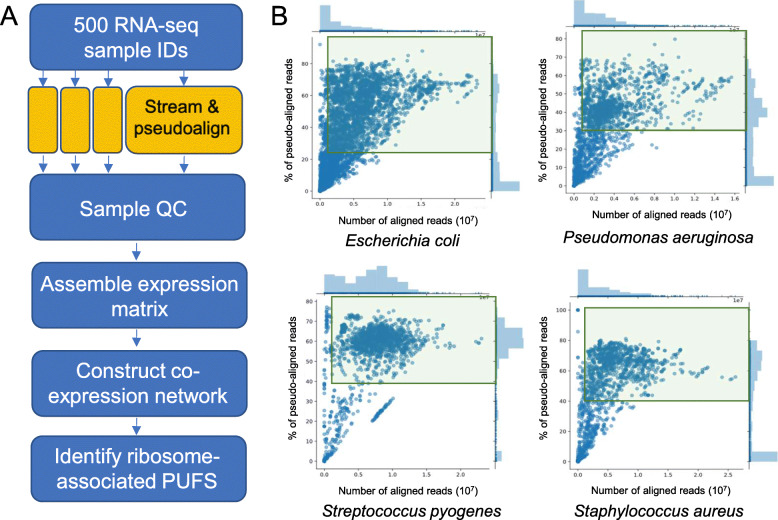
LSTrAP-Crowd pipeline and sample quality control. **a** LSTrAP-Crowd pipeline. The pipeline is a modification of LSTrAP-Cloud, where the modification allows one group of students to share the task streaming and pseudo-aligning the RNA-seq data. **b** Scatter plot showing the number of pseudoaligned reads (n_pseudoaligned, *x*-axis) and % of pseudoaligned reads (p_pseudoaligned, *y*-axis) of RNA-seq samples for four bacteria. Samples with n_pseudoaligned > 1,000,000 and with high p_pseudoaligned values that were not dissimilar from the majority of samples were used to build the expression matrices. Used samples are indicated by green rectangles

The RNA-seq data was streamed by using a modified LSTRaP-Cloud pipeline (Fig. 1a), which gives each user a free Google Colab notebook equipped with a 2 core Xeon CPU and 12 Gb of RAM [47]. The modified pipeline, LSTrAP-Crowd, thus allows a large group of people to download the gene expression data collaboratively. Two hundred eighty-five first-year undergraduate students were divided into 60 groups, with each group tasked to download a maximum of 600 RNA-seq samples (Additional file 1: Table S1). The size of each RNA-seq sample was capped at ~ 1 Gb, allowing a person running the modified LSTRaP-Crowd pipeline to download ~ 300 RNA-seq samples per day [47]. Theoretically, 85,500 (300 × 285) RNA-seq samples equivalent to ~ 85 Tb could be processed per day by the classroom.

**Fig. 2:**
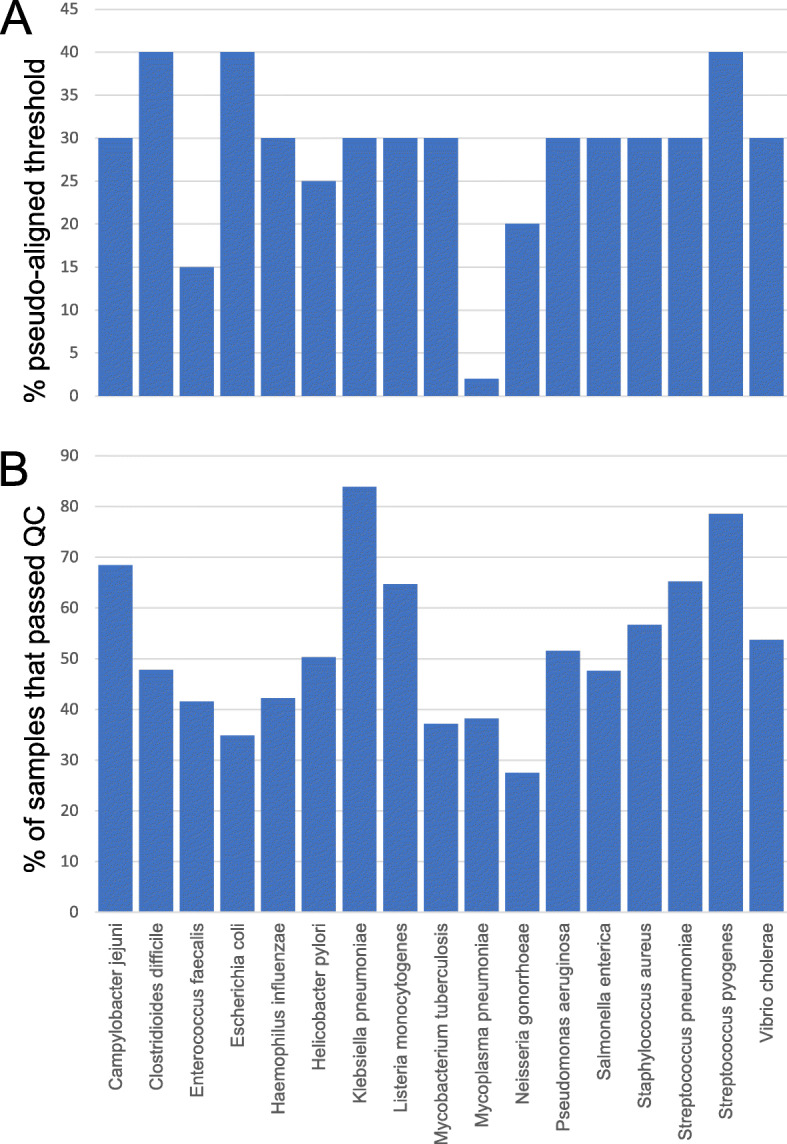
Quality control of the 26,270 RNA-seq samples. **a** % pseudoaligned (p_pseudoaligned) threshold set for the 17 bacteria. **b** The percentage of samples that passed (blue) or failed the p_pseudoaligned and n_pseudoaligned > 1,000,000 thresholds

For each species, all the processed RNA-seq experiments were visualized as scatter plots that show the percentage (*y*-axis) against the number (*x*-label) of reads pseudoaligned to the respective species’ CDS (Fig. 1b). For each experiment, high pseudoalignment percentage indicates high sequence similarity to the CDS, whereas a high absolute number of reads indicates whether the experiment has sufficient data for meaningful coexpression analysis. In this study, a minimum threshold of 1 million reads pseudoaligned was required for the experiment to be considered. We removed samples with n_pseudoaligned < 1,000,000 and with p_pseudoaligned values that were lower than the majority of the high p_pseudoaligned samples (typically > 30%) (Fig. 2a, Additional file 2: Figure S1). The scatterplot pattern was different for each bacteria, most likely due to each bacteria having a different ratio of coding to non-coding DNA (Additional file 2: Figure S1). Samples that passed these thresholds (Fig. 2b) were used to build expression matrices (Additional file 3: Table S2, Additional file 4: Table S3, Additional file 5: Table S4, Additional file 6: Table S5, Additional file 7: Table S6, Additional file 8: Table S7, Additional file 9: Table S8, Additional file 10: Table S9, Additional file 11: Table S10, Additional file 12: Table S11, Additional file 13: Table S12, Additional file 14: Table S13, Additional file 15: Table S14, Additional file 16: Table S15, Additional file 17: Table S16, Additional file 18: Table S17, Additional file 19: Table S18) and used for the co-expression analysis and identification of novel genes involved in protein synthesis.

### Construction and evaluation of co-expression networks for the 17 bacteria

**Fig. 3:**
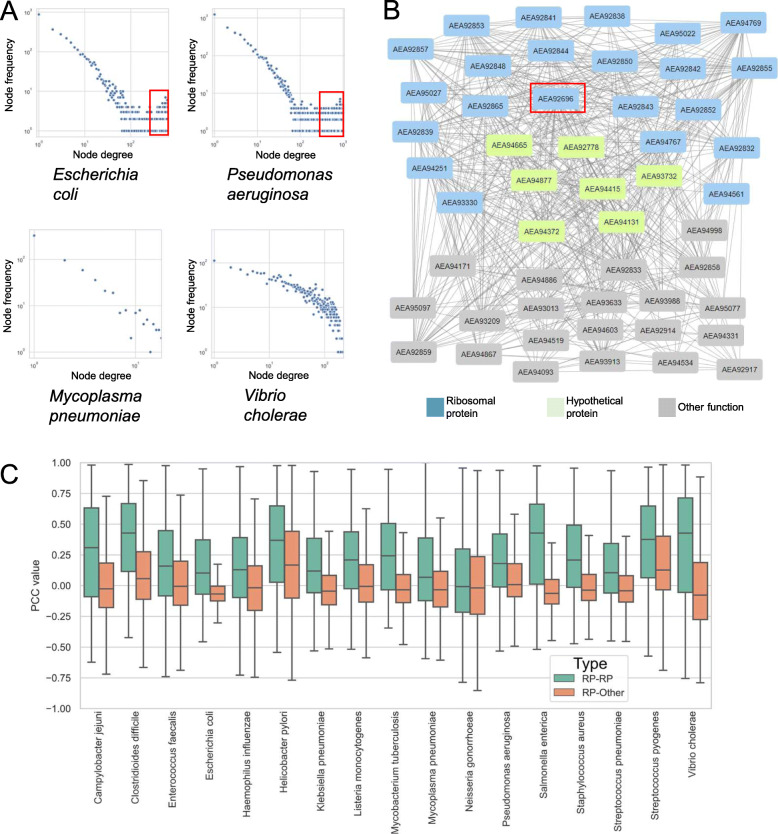
Power-law and example of a ribosomal network. **a** Power-law plot obtained from the expression data of the 17 bacteria. The *x*-axis shows the node degree (number of co-expression connections of a gene), while the *y*-axis indicates the frequency of a degree. Pearson correlation coefficient (PCC) > 0.7 was used to decide whether two genes are co-expressed. The two axes are log10-transformed. **b** Co-expression neighborhood of *AEA92696* from *Enterococcus faecalis* (red square), a 30S ribosomal protein S18, and the 50 most highly co-expressed genes (including *AEA92696*). Nodes indicate genes, while gray edges connect genes with PCC > 0.7. Blue nodes represent ribosomal genes, green nodes represent genes with “Hypothetical protein” in their description, while gray nodes indicate genes with other functions. **c** Distribution of PCC values between ribosomal proteins (RP-RP, green bar) and ribosomal proteins and other genes (RP-Other) in the 17 bacteria. The *x*-axis indicates bacteria, while the *y*-axis indicates the PCC values

A small portion of real-world networks is scale-free [53], including co-expression networks (Mutwil et al., 2010). In scale-free networks, only a few genes are connected (correlated) to many genes, while the majority of genes show only a few connections [54]. Scale-free topology is hypothesized to ensure that the network remains mostly unaltered in case of mutations, and is an evolved property that ensures robustness against perturbations [55]. To demonstrate that the expression data of the 17 bacteria can generate biologically meaningful co-expression networks, we investigated whether the data can produce a typical scale-free network. All of the co-expression networks of the 17 bacteria showed a pattern indicative of scale-free topology, as plotting the number of connections a gene has (node degree) against the frequency of this association produced a negative slope (Fig. 3a). This confirms the scale-free topology of the co-expression networks and suggests that the networks are biologically relevant.

Interestingly, we observed that the power-law plots of some bacteria contain more nodes with a higher degree than expected from a network following power law (Fig. 3a, indicated by red squares). While the basis of this phenomenon is outside of the scope of this publication, we speculate that this is caused by the operon structure of the bacterial genes. Interestingly, certain bacteria, such as *Vibrio cholerae* (Fig. 3a) did not show this pattern (see Additional file 20: Fig. S2 for power law plots for all bacteria). Finally, *Mycoplasma pneumoniae* power-law plot showed a small number of points, indicating that few genes show PCC > 0.7 in this bacteria. This could be attributed to most samples in this bacterium showing worse mapping statistics than the other 16 bacteria (Fig. 2a, Additional file 1: Fig. S1), indicating that perhaps the available CDS for *Mycoplasma* are of poor quality.

To demonstrate that our co-expression networks can be used to predict novel components of ribosomes, we investigated the co-expression neighborhood of *AEA92696*, a 30S ribosomal protein S18 from *Enterococcus faecalis*. The neighborhood was constructed by retrieving the top 50 genes with the highest PCC values to *AEA92696* (Additional file 21: Table S19), where gene pairs with PCC > 0.7 are connected (Fig. 3b). Out of 50 genes, 22 (*n* = 44%) were annotated as a component of the 30S (e.g., S15, S4, S3) or 50S (e.g., L15, L3, L14) ribosomal subunit, indicating that genes in this neighborhood are involved in protein synthesis. Interestingly, 7 genes in the neighborhood are annotated as “hypothetical proteins” (Fig. 3b). Since these genes are found in the neighborhood that is likely to be involved in protein synthesis, we propose that these hypothetical proteins are also involved in protein synthesis in *Enterococcus faecalis.* We observed that ribosomal proteins (RP) show distinctively higher PCC values to other RPs in nearly all bacteria (Fig. 3c, RP-RP), when compared to PCC values between ribosomal proteins and non-ribosomal genes (Fig. 3c, RP-Other). Thus, ribosomal proteins tend to be neighbors to other ribosomal proteins in the co-expression networks.

To identify novel components of protein synthesis with high confidence, we set to identify cutoffs that result in most accurate predictions of ribosomal proteins. The two parameters we investigated are (i) PCC cutoff required to identify co-expressed genes and (ii) the minimum percentage of ribosomal protein neighbors (*n*) required to assign a gene to protein synthesis. To benchmark the performance of the networks at these different cutoffs, each known ribosomal protein was treated as a gene with unknown function, and the ability of the networks to correctly predict the involvement of the ribosomal proteins in protein synthesis was scored. To score the performance, we used F1 score, which is a harmonic mean between precision (true positives/(true positives + false positives)) and recall (true positives/(true positives + false negatives)), where higher F1 score indicates high classification accuracy.

**Fig. 4:**
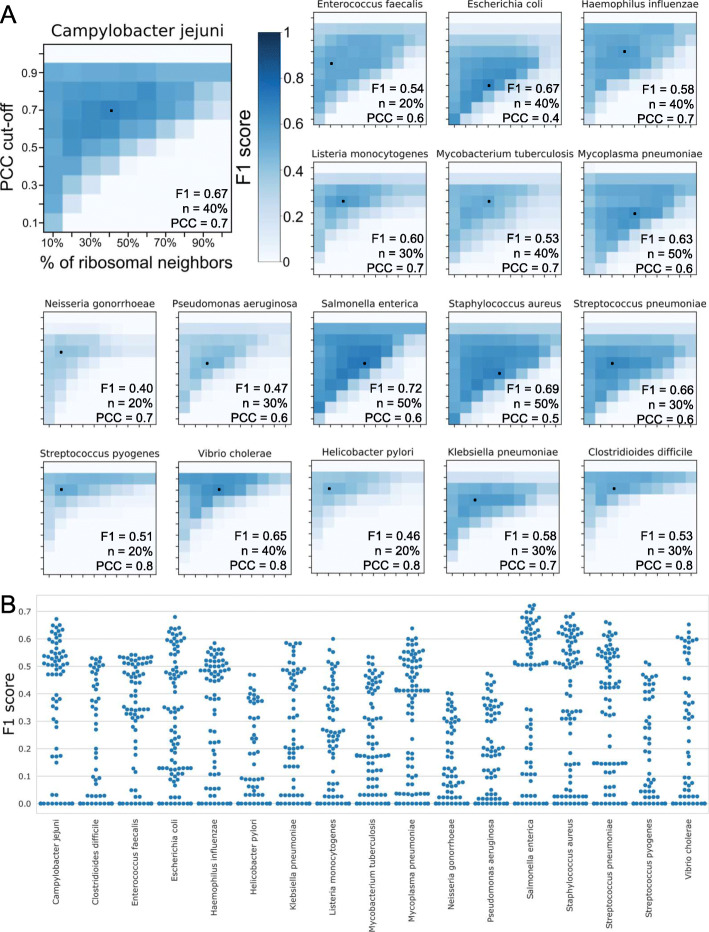
Analysis of thresholds used to predict proteins involved in protein synthesis. **a** The heatmaps indicate the F1 score (harmonic mean of precision and recall) as a function of the percentage of ribosomal protein neighbors (*n* value, *x*-axis) and PCC value (*y*-axis), for the 17 bacteria. The shade of cells in the heatmap indicate low (white) or high (dark blue) F1 score. The highest F1 score for each bacteria is indicated by a black dot, and the percentage of ribosomal protein neighbors (*n*) and PCC value (PCC) are indicated in the lower-right corner of the heatmap. **b** Distribution of the F1 scores for the 17 bacteria. Each dot in the swarmplot indicates an F1 score obtained at PCC values ranging from 0.1 to 1, and *n* values ranging from 10 to 100%

The analysis revealed the PCC cutoff and *n* cutoff combinations that produce the highest F1 score for the 17 bacteria (Fig. 4a). The PCC cutoffs range from > 0.4 (*E. coli*) to > 0.8 (e.g., *S. pyogenes*), while *n* values range from 20% (i.e., > 20% of co-expressed genes should be ribosomal proteins to assign a gene to protein synthesis) to 50%. The F1 score was typically poor at low (0.1) and high (1) PCC cutoffs, as the networks likely connected too many irrelevant (for PCC > 0.1) and no relevant (PCC = 1) genes. Similarly, the *n* value typically resulted in poor F1 score at low (10%) and high (100%) cutoffs, as too many irrelevant genes (10%) or too few relevant genes (100%) were predicted to be involved in protein synthesis. Overall, the maximum F1 scores ranged from ~ 0.4 (*Neisseria gonorrhoeae*) to ~ 0.7 (*Salmonella enterica*).

We compared the performance of our co-expression networks to STRING network database (https://string-db.org/) [56], which integrates genomic neighborhood, gene fusion, genomic co-occurrence, co-expression, experimentally verified function, article text mining, and homology transfer. The comparison revealed that STRING performs similarly to our *E. coli* co-expression networks (Additional file 22: Figure S3, Fig. 4b), while for other bacteria, STRING showed higher F1 scores. This is not surprising, as methods that integrate multiple functional evidences tend to perform better than predictions based on only one evidence, such as co-expression [24, 27]. However, we note that only 4 (*C. jejuni*, *E. coli*, *P. aeruginosa*, and *S. aureus*) out of the 17 bacteria that we used in our analysis contained co-expression networks in STRING, which precludes STRING from using co-expression to identify genes involved in protein synthesis.

### Prediction of novel components of ribosomes by a meta-analysis of the co-expression networks

**Fig. 5:**
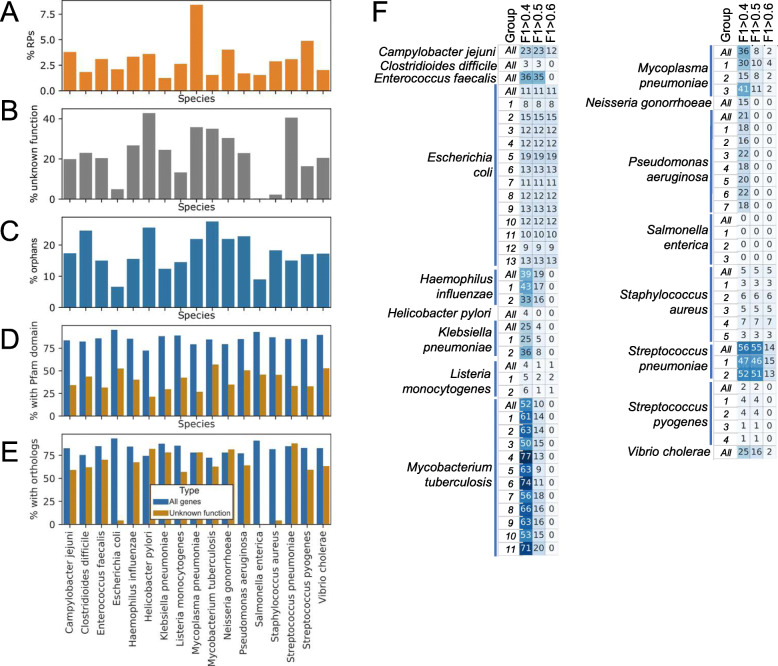
Predicting novel components of ribosomes in the 17 bacteria. **a** The percentage of ribosomal genes in the genomes of the 17 bacteria. **b** The percentage of genes with unknown function. **c** The percentage of genes that do not belong to gene families. **d** The percentage of all genes (blue bars), and genes with unknown function (orange bars) that contain Pfam domains. **e** The percentage of all genes (blue bars) and genes with unknown function (orange bars) that belong to gene families. **f** Number of genes with unknown function that are predicted to be involved in protein synthesis in the 17 bacteria. The predictions made by the 60 groups are shown in rows, and the groups are numbered (e.g., *E. coli* data is divided into 13 groups). Predictions made on all available data are indicated by “All” in the group column. The three columns indicate the F1 score cutoff that was used to assign a gene with unknown function to protein synthesis

To predict which genes with unknown function are involved in protein synthesis in the 17 bacteria, we first identified genes that are involved in protein synthesis (search term “ribosom”) or shared no similarity to any characterized gene (search term “hypothetical,” “DUF,” “conserved”). The analysis revealed that typically, the ribosomal genes constitute < 5% of all genes in a bacterial genome (Fig. 5a). In comparison, the number of genes that are without functional annotation varies from < 1% (*Salmonella enterica*) to 43% (*Helicobacter pylori*) (Fig. 5b). Furthermore, between 7% (*Escherichia coli*) and 23% (*Mycobacterium tuberculosis*) of genes are orphans that do not belong to a gene family (Fig. 5c). Typically, genes with unknown function (orange bars) contain less Pfam domains than characterized genes (Fig. 5d, blue bars), but are frequently found in gene families (Fig. 5e, typically > 50% of genes, orange bars).

To predict uncharacterized genes that are involved in protein synthesis, we applied the same approach that we used to calculate the performance of the networks at different PCC and *n* thresholds. More specifically, for each uncharacterized gene, we calculated the percentage of ribosomal gene neighbors (*n*) at a given PCC cutoff (Additional file 23: Table S20). Since we have calculated the F1 score at the different PCC and *n* thresholds, we could predict which genes are involved in protein synthesis at different (> 0.4, > 0.5, > 0.6) F1 score thresholds. By increasing the F1 score threshold, the prediction can be made more stringent, at the cost of the number of genes with the unknown function assigned to protein synthesis (Fig. 5f, Additional file 24: Table S21).

We observed a varying number of predictions between the different bacteria, ranging from 0 uncharacterized genes assigned to protein synthesis (*Salmonella enterica*) to 77 (*Mycobacterium tuberculosis*, Fig. 5f). As expected, the number of predictions dropped when the F1 score threshold was increased, with few genes assigned to protein synthesis at *F*1 ≥ 0.6 threshold. Interestingly, we observed a good agreement between the numbers of predictions made by different student groups. For example, *Mycobacterium tuberculosis* expression data (6495 samples, Table 1) was divided among 11 student groups and used to perform 11 independent predictions (group 1–11), which we compared to a prediction based on the combined data (all). The prediction based on all data (10 uncharacterized genes assigned to protein synthesis at F1 > 0.5 threshold) did not contain more predicted genes than a subset of the data (e.g., 20 genes at F1 > 0.5, for group 11, 600 samples), indicating that more expression data does not result in more predictions. Furthermore, while each group predicted some unique genes, the majority of the predictions identified the same set of genes (Additional file 24: Table S21), indicating that more data is not necessarily better.

The 17 bacteria showed contrasting protein domain (Additional file 25: Figure S4A) and gene family (Additional file 25: Figure S4B) patterns for the predicted genes. For example, while 100% of the genes belonged to gene families or contained Pfam domains in *E. coli*, this was true for less than 40% of genes in C. jejuni (Additional file 25: Figure S4A). Furthermore, STRING analysis of the 11 *E. coli* genes predicted to be involved in protein synthesis (Fig. 5f, all data, F > 0.6), revealing that 4 of the 11 genes could be associated with protein synthesis by STRING (Additional file 26: Figure S5). Conversely, the other 7 could not be associated with any function by STRING, suggesting that our approach is a valuable addition in gene function prediction in bacteria.

## Discussion

Protein sequence similarity is commonly used to transfer molecular function annotation from one protein to another [57]. Molecular function annotation by sequence comparison is commonly performed using programs such as BLAST [58] and InterProScan [59]. However, a substantial proportion of coding sequences lack sequence similarity to any characterized genes (Fig. 4) [24, 36], making sequence similarity-based inference of gene function unsuitable. An excellent example of this limitation are genes that we have analyzed in this study. Since these genes are annotated as “hypothetical protein,” “domain of unknown function,” or “conserved protein,” they are likely not to share sequence similarity to characterized proteins.

Transcriptomic data is a rapidly growing resource that captures gene expression levels of all genes in an organism. Co-expression analysis is based on the observation that functionally related genes tend to have similar expression profiles across different experiments, and has become a powerful tool for predicting gene function [60]. We applied this approach to identify novel components of protein synthesis machinery in the 17 most notorious bacteria pathogens, for which sufficient (defined as > 100 RNA-seq samples) expression data exists (Table 1). In this study, we achieved two aims.

Firstly, we show co-expression analysis can be used to predict novel candidates of bacterial ribosomes. We observed that ribosomal proteins tend to be strongly co-expressed (Fig. 3b, c), suggesting that uncharacterized genes co-expressed with the ribosomal proteins are likely involved in some aspect (ribosome assembly, protein synthesis, termination) of protein synthesis. We predicted a substantial number of novel genes involved in protein synthesis for 16 out of 17 bacteria (Fig. 5f, Additional file 24: Table S21) that can serve as good targets to develop species-specific antibiotics. The available expression data for the 17 bacteria can be further mined to study other biological functions and vulnerabilities (e.g., cell wall, RNA, and DNA biosynthesis) of these bacteria.

Secondly, we show that such analysis can be outsourced to a large group of individuals. Here, the gene expression data was streamed and pseudo-aligned by 285 first-year undergraduate students, as part of the Computational Thinking class project. To this end, we used a modified LSTrAP-Cloud pipeline [47], where the students were divided into 60 groups, and each group was tasked to download and perform quality-control of ~ 600 samples over a week (Fig. 1 and 2, Additional file 1: Table S1). Theoretically, 85,500 (300 × 285) RNA-seq samples equivalent to ~ 85 Tb could be processed per day by the class, providing a computing power rivaling a high-end computer cluster. While each student had access to only two Xeon cores, one of the major bottlenecks in processing the voluminous RNA-seq data, data download, was circumvented by fast Internet connection of each Google Colab virtual machine. Since each student uses a different virtual machine with an independent Internet connection, our approach demonstrates how a large quantity of data can be analyzed for free.

While similar approaches are used by, e.g., folding@home, it is to our knowledge the first attempt to process gene expression data in such a manner. We envision that similar approaches will soon allow us to study gene expression data within and across whole kingdoms of life.

## Conclusions

To help identify novel components of bacterial ribosomes, we have used co-expression analysis to associate genes with unknown function to ribosomal proteins in 17 pathogenic bacteria. Our analysis identified more than 200 candidates for further functional studies, while our approach exemplified how such analysis can be outsourced to, e.g., a group of undergraduate students.

## Methods

### Streaming RNA sequencing data

The LSTrAP-Crowd pipeline was implemented on Google Colaboratory and is based on the LSTrAP-Cloud pipeline with standard parameters [47]. The pipeline streams the RNA-seq fastq files to a virtual machine in the cloud and deposits the processed gene expression data on the user’s Google Drive. The CDSs were obtained from EnsembleGenomes and used to generate an index file by Kallisto [50], for subsequent estimation of gene expression. The RNA sequencing data of the 17 bacteria was obtained from European Nucleotide Archive (ENA) and mapped against the kallisto index of coding sequences (CDS) of the 17 bacteria. The used CDSs are *Campylobacter jejuni* (Campylobacter_jejuni_subsp_jejuni_cg8421.ASM17179v2.cds.all.fa), *Clostridioides difficile* (Clostridioides_difficile_e25.E25.cds.all.fa), *Enterococcus faecalis* (Enterococcus_faecalis_og1rf.ASM17257v2.cds.all.fa), *Escherichia coli* (Escherichia_coli_str_k_12_substr_mg1655.ASM584v2.cds.all.fa), *Haemophilus influenzae* (Haemophilus_influenzae_r3021.ASM16975v1.cds.all.fa), *Helicobacter pylori* (Helicobacter_pylori_b8.ASM19675v1.cds.all.fa), *Klebsiella pneumoniae* (Klebsiella_pneumoniae_jm45.ASM44540v1.cds.all.fa), *Listeria monocytogenes* (Listeria_monocytogenes_gca_001027125.ASM102712v1.cds.all.fa), *Mycobacterium tuberculosis* (Mycobacterium_tuberculosis_h37rv.ASM19595v2.cds.all.fa), *Mycoplasma pneumoniae* (Mycoplasma_pneumoniae_fh.ASM14394v1.cds.all.fa), *Neisseria gonorrhoeae* (Neisseria_gonorrhoeae_gca_001047275.ASM104727v1.cds.all.fa), *Pseudomonas aeruginosa* (Pseudomonas_aeruginosa_gca_001181725.E11_London_26_VIM_2_06_13.cds.all.fa), *Salmonella enterica* (Salmonella_enterica_subsp_enterica_serovar_typhimurium_str_lt2.ASM694v2.cds.all.fa), *Staphylococcus aureus* (Staphylococcus_aureus_gca_001212685.7738_4_69.cds.all.fa), *Streptococcus pneumoniae* (Streptococcus_pneumoniae_r6.ASM704v1.cds.all.fa), *Streptococcus pyogenes* (Streptococcus_pyogenes_ns88_2.SPNS88.2.cds.all.fa), and *Vibrio cholerae* (Vibrio_cholerae_v51.ASM15246v2.cds.all.fa). All available RNA-sequencing data for the 17 bacteria, comprising different growth conditions, media compositions and mutant strains, were downloaded and subjected to quality control. A total of 26,269 experiments were streamed (Additional file 1: Table S1), where each student used their own Gmail account to connect to their own cloud virtual machine (VM) provided by Google. To coordinate the download effort among the 285 students, the students mounted a Google Drive provided by the instructor. The drive was used to store all of the downloaded data. The main disadvantage of Google Colab VM is that each VM is stopped and erased by Google after 12 h. However, since the processed data is stored in a persistent manner on the shared Google Drive, the interrupted downloads can be easily resumed.

### Generating gene expression matrices for the 17 bacteria

To remove RNA-seq samples that are of lower quality, we identified outlier samples that show a lower number (n_pseudoaligned) and percentage (p_pseudoaligned) of reads aligned to the coding sequences than the majority of the samples. This analysis assumes that the majority of samples are of good quality. The expression matrices containing the gene expression data that passed these thresholds are available in Additional file 3: Table S2, Additional file 4: Table S3, Additional file 5: Table S4, Additional file 6: Table S5, Additional file 7: Table S6, Additional file 8: Table S7, Additional file 9: Table S8, Additional file 10: Table S9, Additional file 11: Table S10, Additional file 12: Table S11, Additional file 13: Table S12, Additional file 14: Table S13, Additional file 15: Table S14, Additional file 16: Table S15, Additional file 17: Table S16, Additional file 18: Table S17, and Additional file 19: Table S18. Additional file 1: Table S1 contains the n_pseudoaligned and p_pseudoaligned numbers and indicates which samples passed the thresholds.

### Identification of genes involved in ribosome biogenesis with co-expression networks

To identify genes that are involved in protein synthesis in the 17 bacteria, we have first retrieved all genes containing “DUF” (domain of unknown function), “hypothetical,” or “conserved” in their description. Next, we calculated the Pearson correlation coefficient (PCC) between the uncharacterized genes and all genes in the genome, where PCC thresholds ranging from 0.1 to 1 were used to indicate co-expressed genes. Finally, the uncharacterized genes were predicted to be involved in protein synthesis if > 10%, >20%, > 30%, >40%, > 50%, >60%, > 70%, >80% or > 90% of the genes were co-expressed with contained annotations such as “ribosome” or “ribosomal.”

### Identification of protein domains and gene families

The protein sequences were obtained from EnsembleGenomes. We used Interproscan-5.44-79 [61] to obtain the Pfam domains. Groups of orthologous genes (gene families) were obtained using Orthofinder v2.3.12 [62] with Diamond [63], with default settings.

## Supplementary information

**Additional file 1 : Table S1.** Quality control of the RNA-seq samples. The table indicates the species (first column), sample ID (second column), group ID processing the sample (third column), number of pseudoaligned reads (fourth column), percentage of pseudoaligned reads (fifth column) and an indication whether the sample passed the set quality thresholds (sixth column).

**Additional file 2 : Figure S1**. Scatter plot showing the number (x-axis) and percentage (y-axis) of pseudoaligned reads for the 17 bacteria.

**Additional file 3 : Table S2.** Campylobacter jejuni expression matrix. Genes are found in rows, while samples are found in columns.

**Additional file 4 : Table S3.** Clostridioides difficile expression matrix. Genes are found in rows, while samples are found in columns.

**Additional file 5 : Table S4.** Enterococcus faecalis expression matrix. Genes are found in rows, while samples are found in columns.

**Additional file 6 : Table S5.** Escherichia coli expression matrix. Genes are found in rows, while samples are found in columns.

**Additional file 7 : Table S6.** Haemophilus influenzae expression matrix. Genes are found in rows, while samples are found in columns.

**Additional file 8 : Table S7.** Helicobacter pylori expression matrix. Genes are found in rows, while samples are found in columns.

**Additional file 9 : Table S8.** Klebsiella pneumoniae expression matrix. Genes are found in rows, while samples are found in columns.

**Additional file 10 : Table S9.** Listeria monocytogenes expression matrix. Genes are found in rows, while samples are found in columns.

**Additional file 11 : Table S10.** Mycobacterium tuberculosis expression matrix. Genes are found in rows, while samples are found in columns.

**Additional file 12 : Table S11.** Mycoplasma pneumoniae expression matrix. Genes are found in rows, while samples are found in columns.

**Additional file 13 : Table S12.** Neisseria gonorrhoeae expression matrix. Genes are found in rows, while samples are found in columns.

**Additional file 14 : Table S13.** Pseudomonas aeruginosa expression matrix. Genes are found in rows, while samples are found in columns.

**Additional file 15 : Table S14.** Salmonella enterica expression matrix. Genes are found in rows, while samples are found in columns.

**Additional file 16 : Table S15.** Staphylococcus aureus expression matrix. Genes are found in rows, while samples are found in columns.

**Additional file 17 : Table S16.** Streptococcus pneumoniae expression matrix. Genes are found in rows, while samples are found in columns.

**Additional file 18 : Table S17.** Streptococcus pyogenes expression matrix. Genes are found in rows, while samples are found in columns.

**Additional file 19 : Table S18**. Vibrio cholerae expression matrix. Genes are found in rows, while samples are found in columns.

**Additional file 20 : Figure S2**. Power-law plot of the 17 bacteria. The x-axis shows the node degree (number of coexpression connections of a gene, PCC > 0.7), while the y-axis indicates the frequency of a degree. The two axes are log10-transformed.

**Additional file 21 : Table S19.** Co-expression neighborhood of *AEA92696* from *Enterococcus faecalis.* The genes are sorted according to the Pearson Correlation Coefficient (r, first column). The gene IDs (second column), type (third column, 1 = ribosomal protein, 2 = gene with unknown function, 0 = not 1 or 2) and annotation (fourth column) are indicated.

**Additional file 22 : Figure S3.** F1 score values for co-function networks obtained from STRING. Distribution of the F1 scores for 4 bacteria. Each dot in the swarmplot indicates an F1 score obtained at PCC values ranging from 0.1 to 1, and n values ranging from 10% to 100%. Only the bacteria for which we could identify common gene identifiers in STRING and our analysis are included.

**Additional file 23 : Table S20.** Gene identifiers of uncharacterized genes predicted to be involved in protein synthesis. The columns indicate the gene ID (first), bacterial species and student group (second), PCC cutoff (third), number of ribosomal protein neighbors at a PCC cutoff (fourth) and number of all gene neighbors at PCC cutoff (fifth).

**Additional file 24 : Table S21.** Uncharacterized genes predicted to be involved in protein synthesis. Each row contains genes with unknown functions predicted to be involved in protein synthesis. The rows contain predictions made by each group (indicated by numbers) or by all available data (All data). The columns indicate the (i) bacteria, (ii) group ID, (iii-vii) predicted genes at the different F1 score thresholds.

**Additional file 25 : Figure S4**. Protein domain and gene family analysis of the genes predicted to be involved in protein synthesis. A) Percentage of genes with Pfam domains. B) Percentage of genes belonging to an orthogroup. The color bars indicate the F1 score threshold used to identify the genes.

**Additional file 26 : Figure S5.** STRING analysis of the 11 genes predicted to be involved in protein synthesis in E.coli by our analysis. The 11 genes are indicated with a red node. Below each network, functional enrichments that are detected by STRING are indicated. *AAC75719*, *AAC74172*, *AAC75326* and *AAC76626* are the four genes significantly associated with genes involved in protein synthesis.

## Data Availability

All data generated or analyzed during this study are included in this published article, its supplementary information files, and publicly available repositories. The RNA-seq data was downloaded from European Nucleotide Archive. The sample identifiers used in this study are found in Additional file 1: Table S1.
