# Supplementary figures and images for: LSTrAP-Crowd: prediction of novel components of bacterial ribosomes with crowd-sourced analysis of RNA sequencing data
Source: BMC Biol. 2020 Sep 3;18:114. doi: 10.1186/s12915-020-00846-9 (PMC7470450; doi:10.1186/s12915-020-00846-9)

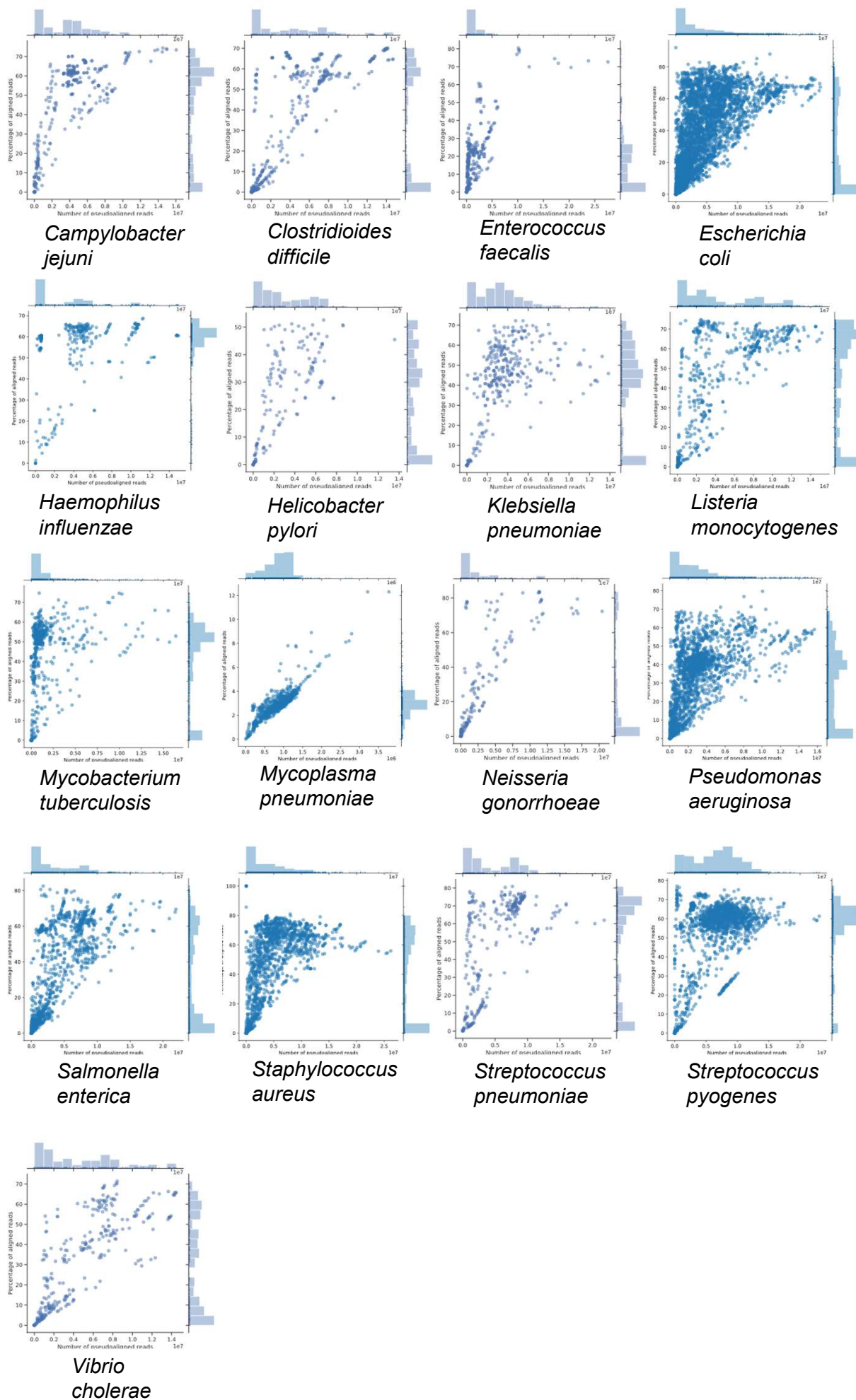

Supplement: Supplementary file 2 — Additional file 2 : Figure S1. Scatter plot showing the number (x-axis) and percentage (y-axis) of pseudoaligned reads for the 17 bacteria. [file 12915_2020_846_MOESM2_ESM.pdf]

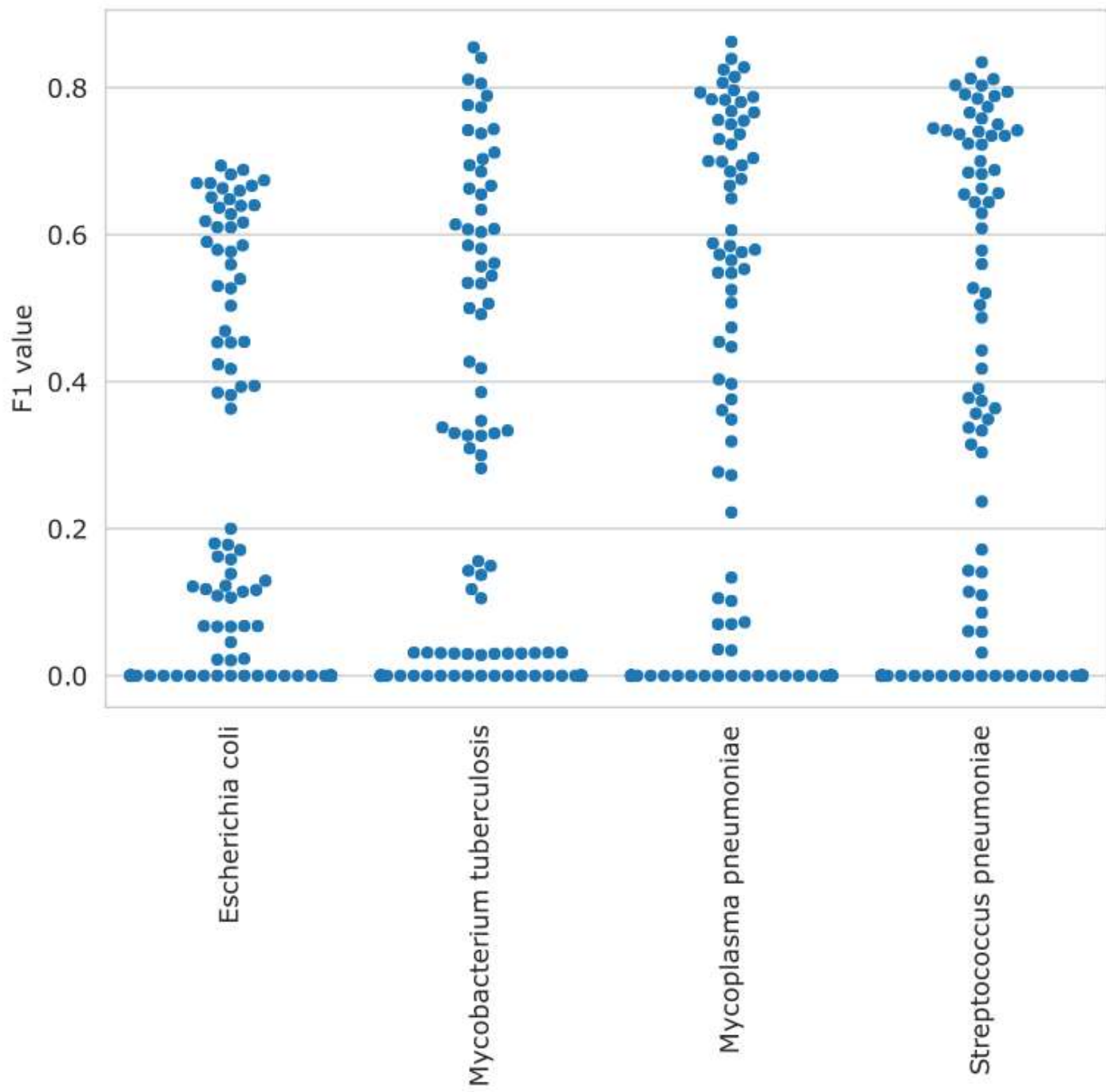

Supplement: Supplementary file 22 — Additional file 22 : Figure S3. F1 score values for co-function networks obtained from STRING. Distribution of the F1 scores for 4 bacteria. Each dot in the swarmplot indicates an F1 score obtained at PCC values ranging from 0.1 to 1, and n values ranging from 10% to 100%. Only the bacteria for which we could identify common gene identifiers in STRING and our analysis are included. [file 12915_2020_846_MOESM22_ESM.pdf]

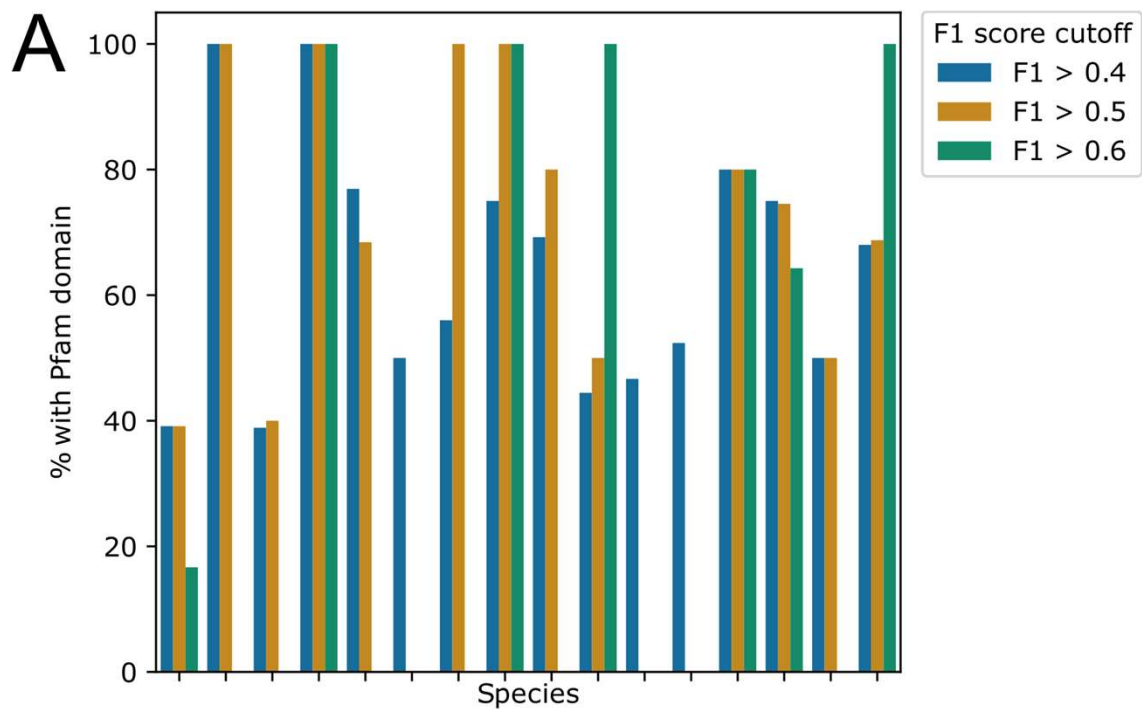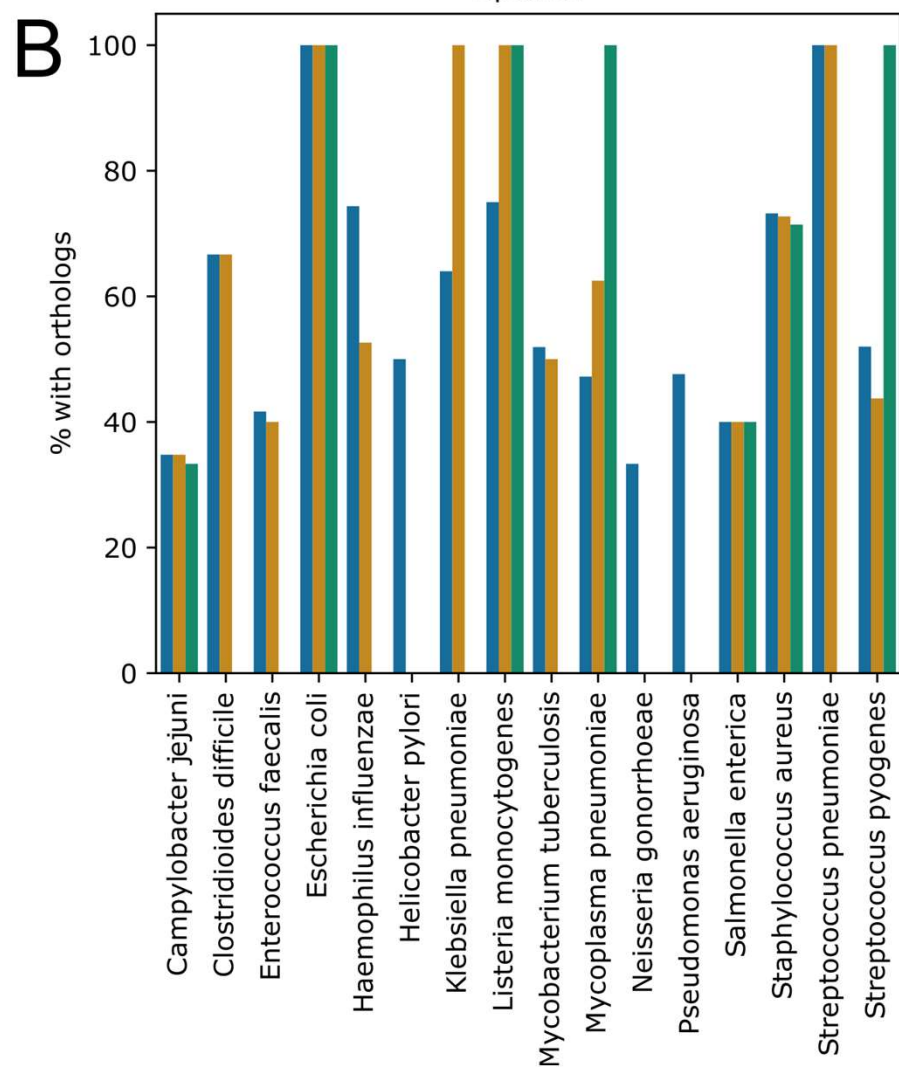

Supplement: Supplementary file 25 — Additional file 25 : Figure S4. Protein domain and gene family analysis of the genes predicted to be involved in protein synthesis. A) Percentage of genes with Pfam domains. B) Percentage of genes belonging to an orthogroup. The color bars indicate the F1 score threshold used to identify the genes. [file 12915_2020_846_MOESM25_ESM.pdf]

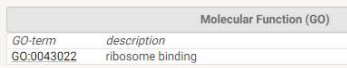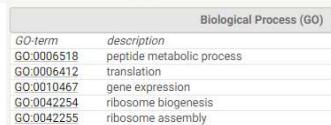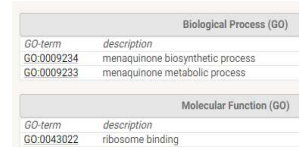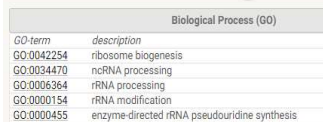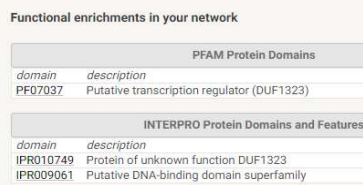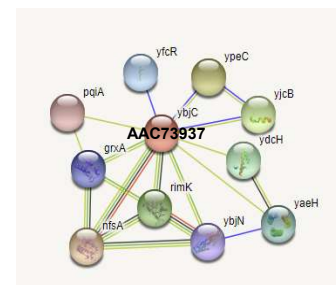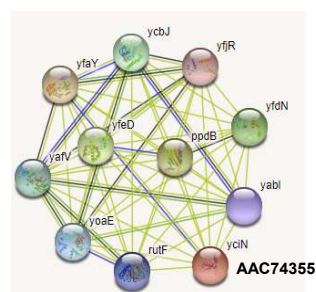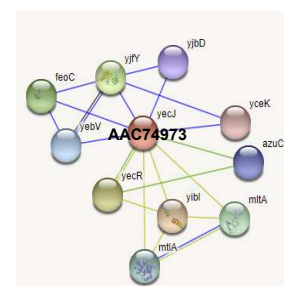

Supplement: Supplementary file 26 — Additional file 26 : Figure S5. STRING analysis of the 11 genes predicted to be involved in protein synthesis in E.coli by our analysis. The 11 genes are indicated with a red node. Below each network, functional enrichments that are detected by STRING are indicated. AAC75719, AAC74172, AAC75326 and AAC76626 are the four genes significantly associated with genes involved in protein synthesis. [file 12915_2020_846_MOESM26_ESM.pdf]
