# Supplementary material for: LSTrAP-Crowd: prediction of novel components of bacterial ribosomes with crowd-sourced analysis of RNA sequencing data
Source: BMC Biol. 2020 Sep 3;18:114. doi: 10.1186/s12915-020-00846-9 (PMC7470450; doi:10.1186/s12915-020-00846-9)
Supplement: Supplementary file 20 — Additional file 20 : Figure S2. Power-law plot of the 17 bacteria. The x-axis shows the node degree (number of coexpression connections of a gene, PCC > 0.7), while the y-axis indicates the frequency of a degree. The two axes are log10-transformed. [file 12915_2020_846_MOESM20_ESM.pdf]

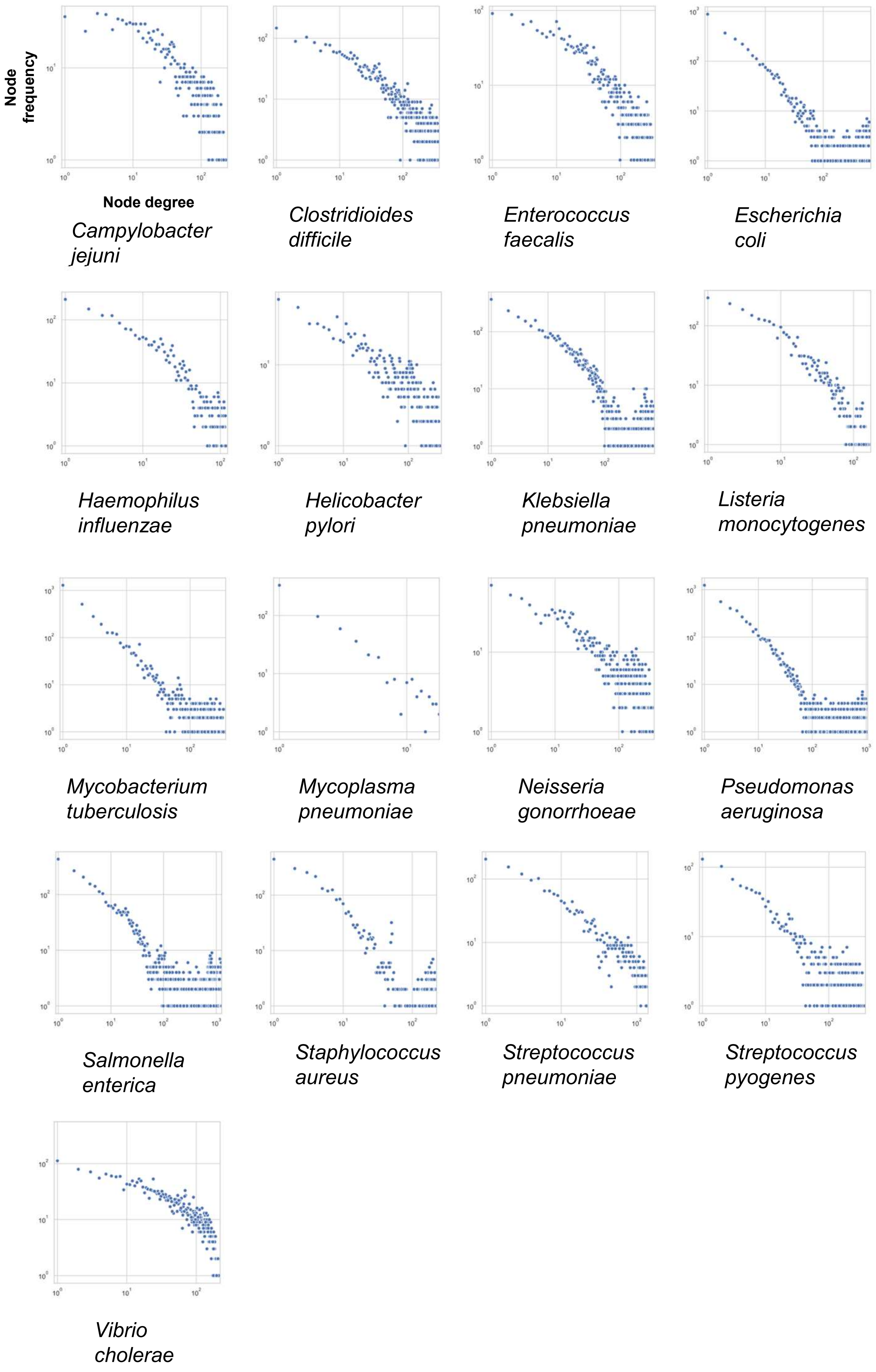
Node frequency
Node degree
Campylobacter jejuni
Clostridioides difficile
Enterococcus faecalis
Escherichia coli
Haemophilus influenzae
Helicobacter pylori
Klebsiella pneumoniae
Listeria monocytogenes
Mycobacterium tuberculosis
Mycoplasma pneumoniae
Neisseria gonorrhoeae
Pseudomonas aeruginosa
Salmonella enterica
Staphylococcus aureus
Streptococcus pneumoniae
Streptococcus pyogenes
Vibrio cholerae
